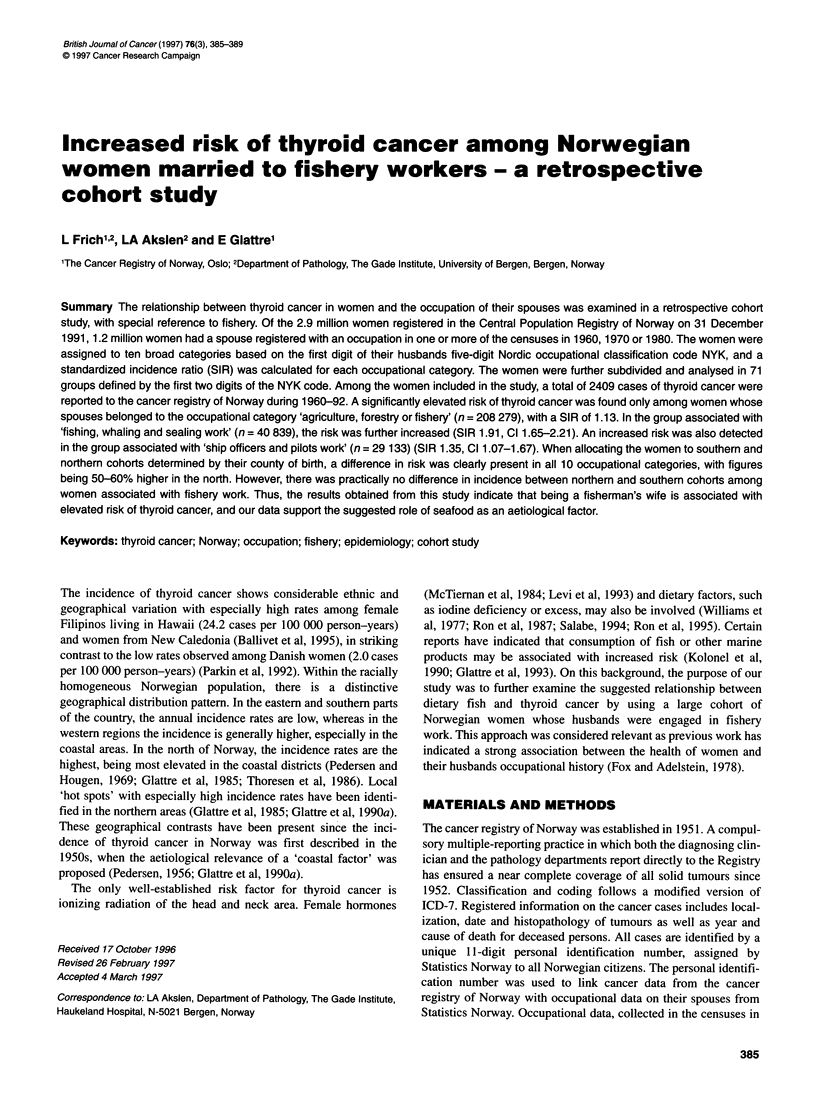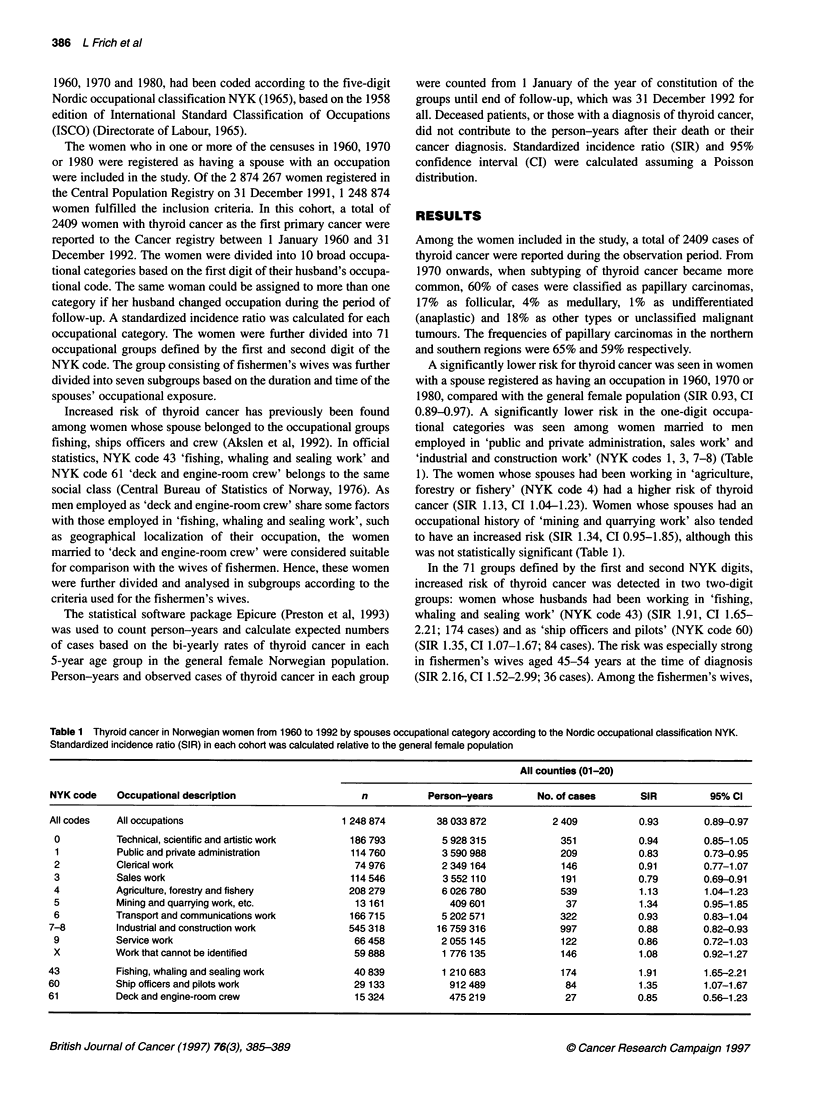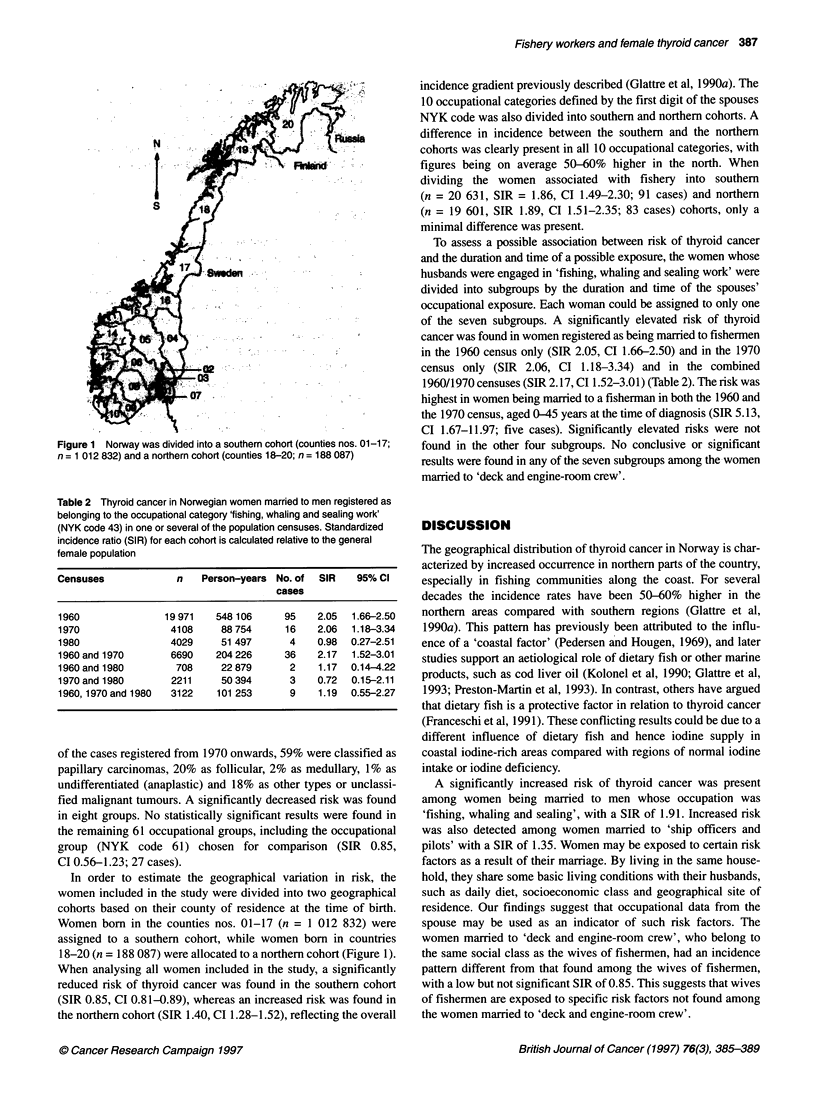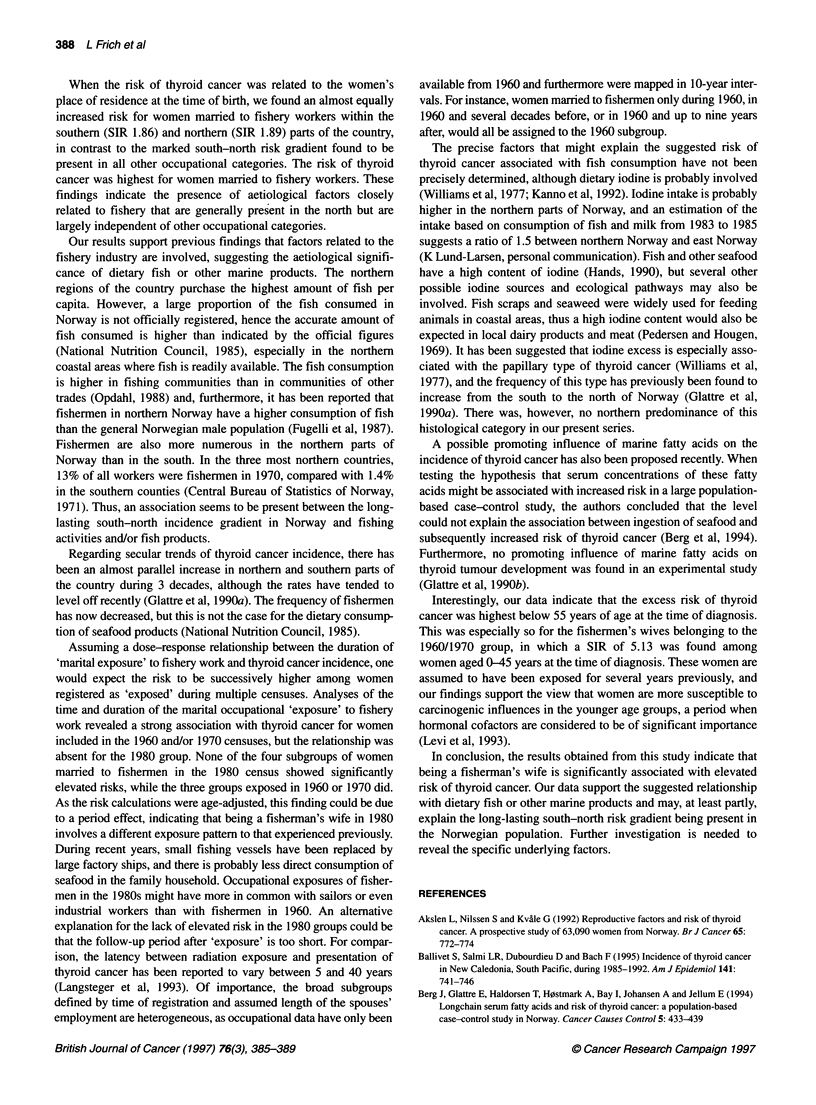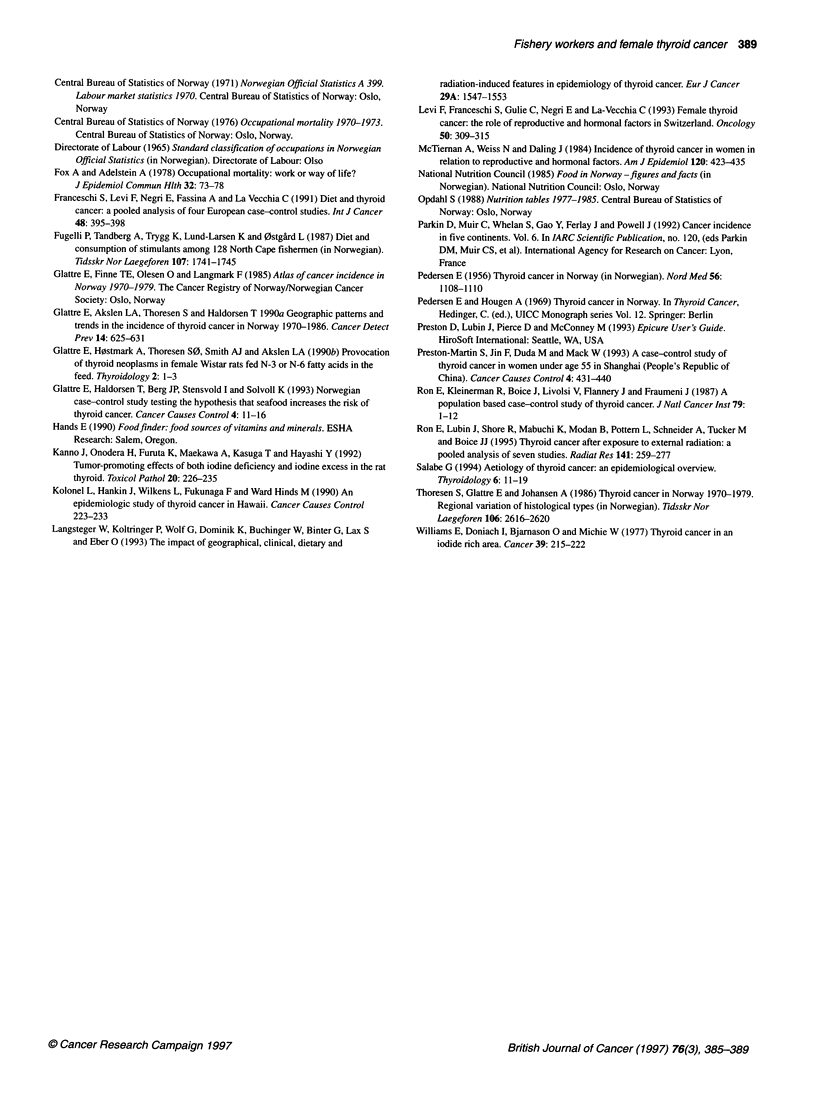# Increased risk of thyroid cancer among Norwegian women married to fishery workers--a retrospective cohort study.

**DOI:** 10.1038/bjc.1997.395

**Published:** 1997

**Authors:** L. Frich, L. A. Akslen, E. Glattre

**Affiliations:** The Cancer Registry of Norway, Oslo.

## Abstract

The relationship between thyroid cancer in women and the occupation of their spouses was examined in a retrospective cohort study, with special reference to fishery. Of the 2.9 million women registered in the Central Population Registry of Norway on 31 December 1991, 1.2 million women had a spouse registered with an occupation in one or more of the censuses in 1960, 1970 or 1980. The women were assigned to ten broad categories based on the first digit of their husbands five-digit Nordic occupational classification code NYK, and a standardized incidence ratio (SIR) was calculated for each occupational category. The women were further subdivided and analysed in 71 groups defined by the first two digits of the NYK code. Among the women included in the study, a total of 2409 cases of thyroid cancer were reported to the cancer registry of Norway during 1960-92. A significantly elevated risk of thyroid cancer was found only among women whose spouses belonged to the occupational category 'agriculture, forestry or fishery' (n = 208 279), with a SIR of 1.13. In the group associated with 'fishing, whaling and sealing work' (n = 40 839), the risk was further increased (SIR 1.91, CI 1.65-2.21). An increased risk was also detected in the group associated with 'ship officers and pilots work' (n = 29 133) (SIR 1.35, CI 1.07-1.67). When allocating the women to southern and northern cohorts determined by their county of birth, a difference in risk was clearly present in all 10 occupational categories, with figures being 50-60% higher in the north. However, there was practically no difference in incidence between northern and southern cohorts among women associated with fishery work. Thus, the results obtained from this study indicate that being a fisherman's wife is associated with elevated risk of thyroid cancer, and our data support the suggested role of seafood as an aetiological factor.


British Journal of Cancer (1997) 76(3), 385–389
© 1997 Cancer Research Campaign

### Increased risk of thyroid cancer among Norwegian women married to fishery workers – a retrospective cohort study

L Frich[1,2], LA Akslen[2] and E Glattre[1]

[1]The Cancer Registry of Norway, Oslo; [2]Department of Pathology, The Gade Institute, University of Bergen, Bergen, Norway

**Summary** The relationship between thyroid cancer in women and the occupation of their spouses was examined in a retrospective cohort study, with special reference to fishery. Of the 2.9 million women registered in the Central Population Registry of Norway on 31 December 1991, 1.2 million women had a spouse registered with an occupation in one or more of the censuses in 1960, 1970 or 1980. The women were assigned to ten broad categories based on the first digit of their husbands five-digit Nordic occupational classification code NYK, and a standardized incidence ratio (SIR) was calculated for each occupational category. The women were further subdivided and analysed in 71 groups defined by the first two digits of the NYK code. Among the women included in the study, a total of 2409 cases of thyroid cancer were reported to the cancer registry of Norway during 1960–92. A significantly elevated risk of thyroid cancer was found only among women whose spouses belonged to the occupational category 'agriculture, forestry or fishery' ($n$ = 208 279), with a SIR of 1.13. In the group associated with 'fishing, whaling and sealing work' ($n$ = 40 839), the risk was further increased (SIR 1.91, CI 1.65–2.21). An increased risk was also detected in the group associated with 'ship officers and pilots work' ($n$ = 29 133) (SIR 1.35, CI 1.07–1.67). When allocating the women to southern and northern cohorts determined by their county of birth, a difference in risk was clearly present in all 10 occupational categories, with figures being 50–60% higher in the north. However, there was practically no difference in incidence between northern and southern cohorts among women associated with fishery work. Thus, the results obtained from this study indicate that being a fisherman's wife is associated with elevated risk of thyroid cancer, and our data support the suggested role of seafood as an aetiological factor.

**Keywords:** thyroid cancer; Norway; occupation; fishery; epidemiology; cohort study

The incidence of thyroid cancer shows considerable ethnic and geographical variation with especially high rates among female Filipinos living in Hawaii (24.2 cases per 100 000 person–years) and women from New Caledonia (Ballivet et al, 1995), in striking contrast to the low rates observed among Danish women (2.0 cases per 100 000 person–years) (Parkin et al, 1992). Within the racially homogeneous Norwegian population, there is a distinctive geographical distribution pattern. In the eastern and southern parts of the country, the annual incidence rates are low, whereas in the western regions the incidence is generally higher, especially in the coastal areas. In the north of Norway, the incidence rates are the highest, being most elevated in the coastal districts (Pedersen and Hougen, 1969; Glattre et al, 1985; Thoresen et al, 1986). Local 'hot spots' with especially high incidence rates have been identified in the northern areas (Glattre et al, 1985; Glattre et al, 1990*a*). These geographical contrasts have been present since the incidence of thyroid cancer in Norway was first described in the 1950s, when the aetiological relevance of a 'coastal factor' was proposed (Pedersen, 1956; Glattre et al, 1990*a*).

The only well-established risk factor for thyroid cancer is ionizing radiation of the head and neck area. Female hormones (McTiernan et al, 1984; Levi et al, 1993) and dietary factors, such as iodine deficiency or excess, may also be involved (Williams et al, 1977; Ron et al, 1987; Salabe, 1994; Ron et al, 1995). Certain reports have indicated that consumption of fish or other marine products may be associated with increased risk (Kolonel et al, 1990; Glattre et al, 1993). On this background, the purpose of our study was to further examine the suggested relationship between dietary fish and thyroid cancer by using a large cohort of Norwegian women whose husbands were engaged in fishery work. This approach was considered relevant as previous work has indicated a strong association between the health of women and their husbands occupational history (Fox and Adelstein, 1978).

*Received 17 October 1996*
*Revised 26 February 1997*
*Accepted 4 March 1997*

*Correspondence to:* LA Akslen, Department of Pathology, The Gade Institute, Haukeland Hospital, N-5021 Bergen, Norway

### MATERIALS AND METHODS

The cancer registry of Norway was established in 1951. A compulsory multiple-reporting practice in which both the diagnosing clinician and the pathology departments report directly to the Registry has ensured a near complete coverage of all solid tumours since 1952. Classification and coding follows a modified version of ICD-7. Registered information on the cancer cases includes localization, date and histopathology of tumours as well as year and cause of death for deceased persons. All cases are identified by a unique 11-digit personal identification number, assigned by Statistics Norway to all Norwegian citizens. The personal identification number was used to link cancer data from the cancer registry of Norway with occupational data on their spouses from Statistics Norway. Occupational data, collected in the censuses in

1960, 1970 and 1980, had been coded according to the five-digit Nordic occupational classification NYK (1965), based on the 1958 edition of International Standard Classification of Occupations (ISCO) (Directorate of Labour, 1965).

The women who in one or more of the censuses in 1960, 1970 or 1980 were registered as having a spouse with an occupation were included in the study. Of the 2 874 267 women registered in the Central Population Registry on 31 December 1991, 1 248 874 women fulfilled the inclusion criteria. In this cohort, a total of 2409 women with thyroid cancer as the first primary cancer were reported to the Cancer registry between 1 January 1960 and 31 December 1992. The women were divided into 10 broad occupational categories based on the first digit of their husband's occupational code. The same woman could be assigned to more than one category if her husband changed occupation during the period of follow-up. A standardized incidence ratio was calculated for each occupational category. The women were further divided into 71 occupational groups defined by the first and second digit of the NYK code. The group consisting of fishermen's wives was further divided into seven subgroups based on the duration and time of the spouses' occupational exposure.

Increased risk of thyroid cancer has previously been found among women whose spouse belonged to the occupational groups fishing, ships officers and crew (Akslen et al, 1992). In official statistics, NYK code 43 'fishing, whaling and sealing work' and NYK code 61 'deck and engine-room crew' belongs to the same social class (Central Bureau of Statistics of Norway, 1976). As men employed as 'deck and engine-room crew' share some factors with those employed in 'fishing, whaling and sealing work', such as geographical localization of their occupation, the women married to 'deck and engine-room crew' were considered suitable for comparison with the wives of fishermen. Hence, these women were further divided and analysed in subgroups according to the criteria used for the fishermen's wives.

The statistical software package Epicure (Preston et al, 1993) was used to count person–years and calculate expected numbers of cases based on the bi-yearly rates of thyroid cancer in each 5-year age group in the general female Norwegian population. Person–years and observed cases of thyroid cancer in each group were counted from 1 January of the year of constitution of the groups until end of follow-up, which was 31 December 1992 for all. Deceased patients, or those with a diagnosis of thyroid cancer, did not contribute to the person–years after their death or their cancer diagnosis. Standardized incidence ratio (SIR) and 95% confidence interval (CI) were calculated assuming a Poisson distribution.

### RESULTS

Among the women included in the study, a total of 2409 cases of thyroid cancer were reported during the observation period. From 1970 onwards, when subtyping of thyroid cancer became more common, 60% of cases were classified as papillary carcinomas, 17% as follicular, 4% as medullary, 1% as undifferentiated (anaplastic) and 18% as other types or unclassified malignant tumours. The frequencies of papillary carcinomas in the northern and southern regions were 65% and 59% respectively.

A significantly lower risk for thyroid cancer was seen in women with a spouse registered as having an occupation in 1960, 1970 or 1980, compared with the general female population (SIR 0.93, CI 0.89–0.97). A significantly lower risk in the one-digit occupational categories was seen among women married to men employed in 'public and private administration, sales work' and 'industrial and construction work' (NYK codes 1, 3, 7–8) (Table 1). The women whose spouses had been working in 'agriculture, forestry or fishery' (NYK code 4) had a higher risk of thyroid cancer (SIR 1.13, CI 1.04–1.23). Women whose spouses had an occupational history of 'mining and quarrying work' also tended to have an increased risk (SIR 1.34, CI 0.95–1.85), although this was not statistically significant (Table 1).

In the 71 groups defined by the first and second NYK digits, increased risk of thyroid cancer was detected in two two-digit groups: women whose husbands had been working in 'fishing, whaling and sealing work' (NYK code 43) (SIR 1.91, CI 1.65–2.21; 174 cases) and as 'ship officers and pilots' (NYK code 60) (SIR 1.35, CI 1.07–1.67; 84 cases). The risk was especially strong in fishermen's wives aged 45–54 years at the time of diagnosis (SIR 2.16, CI 1.52–2.99; 36 cases). Among the fishermen's wives,

**Table 1** Thyroid cancer in Norwegian women from 1960 to 1992 by spouses occupational category according to the Nordic occupational classification NYK. Standardized incidence ratio (SIR) in each cohort was calculated relative to the general female population

| | | All counties (01–20) | | | | |
|---|---|---|---|---|---|---|
| NYK code | Occupational description | *n* | Person–years | No. of cases | SIR | 95% CI |
| All codes | All occupations | 1 248 874 | 38 033 872 | 2 409 | 0.93 | 0.89–0.97 |
| 0 | Technical, scientific and artistic work | 186 793 | 5 928 315 | 351 | 0.94 | 0.85–1.05 |
| 1 | Public and private administration | 114 760 | 3 590 988 | 209 | 0.83 | 0.73–0.95 |
| 2 | Clerical work | 74 976 | 2 349 164 | 146 | 0.91 | 0.77–1.07 |
| 3 | Sales work | 114 546 | 3 552 110 | 191 | 0.79 | 0.69–0.91 |
| 4 | Agriculture, forestry and fishery | 208 279 | 6 026 780 | 539 | 1.13 | 1.04–1.23 |
| 5 | Mining and quarrying work, etc. | 13 161 | 409 601 | 37 | 1.34 | 0.95–1.85 |
| 6 | Transport and communications work | 166 715 | 5 202 571 | 322 | 0.93 | 0.83–1.04 |
| 7–8 | Industrial and construction work | 545 318 | 16 759 316 | 997 | 0.88 | 0.82–0.93 |
| 9 | Service work | 66 458 | 2 055 145 | 122 | 0.86 | 0.72–1.03 |
| X | Work that cannot be identified | 59 888 | 1 776 135 | 146 | 1.08 | 0.92–1.27 |
| 43 | Fishing, whaling and sealing work | 40 839 | 1 210 683 | 174 | 1.91 | 1.65–2.21 |
| 60 | Ship officers and pilots work | 29 133 | 912 489 | 84 | 1.35 | 1.07–1.67 |
| 61 | Deck and engine-room crew | 15 324 | 475 219 | 27 | 0.85 | 0.56–1.23 |

 © Cancer Research Campaign 1997

**Figure 1** Norway was divided into a southern cohort (counties nos. 01–17; $n$ = 1 012 832) and a northern cohort (counties 18–20; $n$ = 188 087)

**Table 2** Thyroid cancer in Norwegian women married to men registered as belonging to the occupational category 'fishing, whaling and sealing work' (NYK code 43) in one or several of the population censuses. Standardized incidence ratio (SIR) for each cohort is calculated relative to the general female population

| Censuses | *n* | Person-years | No. of cases | SIR | 95% CI |
|---|---|---|---|---|---|
| 1960 | 19 971 | 548 106 | 95 | 2.05 | 1.66–2.50 |
| 1970 | 4108 | 88 754 | 16 | 2.06 | 1.18–3.34 |
| 1980 | 4029 | 51 497 | 4 | 0.98 | 0.27–2.51 |
| 1960 and 1970 | 6690 | 204 226 | 36 | 2.17 | 1.52–3.01 |
| 1960 and 1980 | 708 | 22 879 | 2 | 1.17 | 0.14–4.22 |
| 1970 and 1980 | 2211 | 50 394 | 3 | 0.72 | 0.15–2.11 |
| 1960, 1970 and 1980 | 3122 | 101 253 | 9 | 1.19 | 0.55–2.27 |

of the cases registered from 1970 onwards, 59% were classified as papillary carcinomas, 20% as follicular, 2% as medullary, 1% as undifferentiated (anaplastic) and 18% as other types or unclassified malignant tumours. A significantly decreased risk was found in eight groups. No statistically significant results were found in the remaining 61 occupational groups, including the occupational group (NYK code 61) chosen for comparison (SIR 0.85, CI 0.56–1.23; 27 cases).

In order to estimate the geographical variation in risk, the women included in the study were divided into two geographical cohorts based on their county of residence at the time of birth. Women born in the counties nos. 01–17 ($n$ = 1 012 832) were assigned to a southern cohort, while women born in countries 18–20 ($n$ = 188 087) were allocated to a northern cohort (Figure 1). When analysing all women included in the study, a significantly reduced risk of thyroid cancer was found in the southern cohort (SIR 0.85, CI 0.81–0.89), whereas an increased risk was found in the northern cohort (SIR 1.40, CI 1.28–1.52), reflecting the overall incidence gradient previously described (Glattre et al, 1990*a*). The 10 occupational categories defined by the first digit of the spouses NYK code was also divided into southern and northern cohorts. A difference in incidence between the southern and the northern cohorts was clearly present in all 10 occupational categories, with figures being on average 50–60% higher in the north. When dividing the women associated with fishery into southern ($n$ = 20 631, SIR = 1.86, CI 1.49–2.30; 91 cases) and northern ($n$ = 19 601, SIR 1.89, CI 1.51–2.35; 83 cases) cohorts, only a minimal difference was present.

To assess a possible association between risk of thyroid cancer and the duration and time of a possible exposure, the women whose husbands were engaged in 'fishing, whaling and sealing work' were divided into subgroups by the duration and time of the spouses' occupational exposure. Each woman could be assigned to only one of the seven subgroups. A significantly elevated risk of thyroid cancer was found in women registered as being married to fishermen in the 1960 census only (SIR 2.05, CI 1.66–2.50) and in the 1970 census only (SIR 2.06, CI 1.18–3.34) and in the combined 1960/1970 censuses (SIR 2.17, CI 1.52–3.01) (Table 2). The risk was highest in women being married to a fisherman in both the 1960 and the 1970 census, aged 0–45 years at the time of diagnosis (SIR 5.13, CI 1.67–11.97; five cases). Significantly elevated risks were not found in the other four subgroups. No conclusive or significant results were found in any of the seven subgroups among the women married to 'deck and engine-room crew'.

### DISCUSSION

The geographical distribution of thyroid cancer in Norway is characterized by increased occurrence in northern parts of the country, especially in fishing communities along the coast. For several decades the incidence rates have been 50–60% higher in the northern areas compared with southern regions (Glattre et al, 1990*a*). This pattern has previously been attributed to the influence of a 'coastal factor' (Pedersen and Hougen, 1969), and later studies support an aetiological role of dietary fish or other marine products, such as cod liver oil (Kolonel et al, 1990; Glattre et al, 1993; Preston-Martin et al, 1993). In contrast, others have argued that dietary fish is a protective factor in relation to thyroid cancer (Franceschi et al, 1991). These conflicting results could be due to a different influence of dietary fish and hence iodine supply in coastal iodine-rich areas compared with regions of normal iodine intake or iodine deficiency.

A significantly increased risk of thyroid cancer was present among women being married to men whose occupation was 'fishing, whaling and sealing', with a SIR of 1.91. Increased risk was also detected among women married to 'ship officers and pilots' with a SIR of 1.35. Women may be exposed to certain risk factors as a result of their marriage. By living in the same household, they share some basic living conditions with their husbands, such as daily diet, socioeconomic class and geographical site of residence. Our findings suggest that occupational data from the spouse may be used as an indicator of such risk factors. The women married to 'deck and engine-room crew', who belong to the same social class as the wives of fishermen, had an incidence pattern different from that found among the wives of fishermen, with a low but not significant SIR of 0.85. This suggests that wives of fishermen are exposed to specific risk factors not found among the women married to 'deck and engine-room crew'.

© Cancer Research Campaign 1997 

When the risk of thyroid cancer was related to the women's place of residence at the time of birth, we found an almost equally increased risk for women married to fishery workers within the southern (SIR 1.86) and northern (SIR 1.89) parts of the country, in contrast to the marked south–north risk gradient found to be present in all other occupational categories. The risk of thyroid cancer was highest for women married to fishery workers. These findings indicate the presence of aetiological factors closely related to fishery that are generally present in the north but are largely independent of other occupational categories.

Our results support previous findings that factors related to the fishery industry are involved, suggesting the aetiological significance of dietary fish or other marine products. The northern regions of the country purchase the highest amount of fish per capita. However, a large proportion of the fish consumed in Norway is not officially registered, hence the accurate amount of fish consumed is higher than indicated by the official figures (National Nutrition Council, 1985), especially in the northern coastal areas where fish is readily available. The fish consumption is higher in fishing communities than in communities of other trades (Opdahl, 1988) and, furthermore, it has been reported that fishermen in northern Norway have a higher consumption of fish than the general Norwegian male population (Fugelli et al, 1987). Fishermen are also more numerous in the northern parts of Norway than in the south. In the three most northern countries, 13% of all workers were fishermen in 1970, compared with 1.4% in the southern counties (Central Bureau of Statistics of Norway, 1971). Thus, an association seems to be present between the long-lasting south–north incidence gradient in Norway and fishing activities and/or fish products.

Regarding secular trends of thyroid cancer incidence, there has been an almost parallel increase in northern and southern parts of the country during 3 decades, although the rates have tended to level off recently (Glattre et al, 1990*a*). The frequency of fishermen has now decreased, but this is not the case for the dietary consumption of seafood products (National Nutrition Council, 1985).

Assuming a dose–response relationship between the duration of 'marital exposure' to fishery work and thyroid cancer incidence, one would expect the risk to be successively higher among women registered as 'exposed' during multiple censuses. Analyses of the time and duration of the marital occupational 'exposure' to fishery work revealed a strong association with thyroid cancer for women included in the 1960 and/or 1970 censuses, but the relationship was absent for the 1980 group. None of the four subgroups of women married to fishermen in the 1980 census showed significantly elevated risks, while the three groups exposed in 1960 or 1970 did. As the risk calculations were age-adjusted, this finding could be due to a period effect, indicating that being a fisherman's wife in 1980 involves a different exposure pattern to that experienced previously. During recent years, small fishing vessels have been replaced by large factory ships, and there is probably less direct consumption of seafood in the family household. Occupational exposures of fishermen in the 1980s might have more in common with sailors or even industrial workers than with fishermen in 1960. An alternative explanation for the lack of elevated risk in the 1980 groups could be that the follow-up period after 'exposure' is too short. For comparison, the latency between radiation exposure and presentation of thyroid cancer has been reported to vary between 5 and 40 years (Langsteger et al, 1993). Of importance, the broad subgroups defined by time of registration and assumed length of the spouses' employment are heterogeneous, as occupational data have only been available from 1960 and furthermore were mapped in 10-year intervals. For instance, women married to fishermen only during 1960, in 1960 and several decades before, or in 1960 and up to nine years after, would all be assigned to the 1960 subgroup.

The precise factors that might explain the suggested risk of thyroid cancer associated with fish consumption have not been precisely determined, although dietary iodine is probably involved (Williams et al, 1977; Kanno et al, 1992). Iodine intake is probably higher in the northern parts of Norway, and an estimation of the intake based on consumption of fish and milk from 1983 to 1985 suggests a ratio of 1.5 between northern Norway and east Norway (K Lund-Larsen, personal communication). Fish and other seafood have a high content of iodine (Hands, 1990), but several other possible iodine sources and ecological pathways may also be involved. Fish scraps and seaweed were widely used for feeding animals in coastal areas, thus a high iodine content would also be expected in local dairy products and meat (Pedersen and Hougen, 1969). It has been suggested that iodine excess is especially associated with the papillary type of thyroid cancer (Williams et al, 1977), and the frequency of this type has previously been found to increase from the south to the north of Norway (Glattre et al, 1990*a*). There was, however, no northern predominance of this histological category in our present series.

A possible promoting influence of marine fatty acids on the incidence of thyroid cancer has also been proposed recently. When testing the hypothesis that serum concentrations of these fatty acids might be associated with increased risk in a large population-based case–control study, the authors concluded that the level could not explain the association between ingestion of seafood and subsequently increased risk of thyroid cancer (Berg et al, 1994). Furthermore, no promoting influence of marine fatty acids on thyroid tumour development was found in an experimental study (Glattre et al, 1990*b*).

Interestingly, our data indicate that the excess risk of thyroid cancer was highest below 55 years of age at the time of diagnosis. This was especially so for the fishermen's wives belonging to the 1960/1970 group, in which a SIR of 5.13 was found among women aged 0–45 years at the time of diagnosis. These women are assumed to have been exposed for several years previously, and our findings support the view that women are more susceptible to carcinogenic influences in the younger age groups, a period when hormonal cofactors are considered to be of significant importance (Levi et al, 1993).

In conclusion, the results obtained from this study indicate that being a fisherman's wife is significantly associated with elevated risk of thyroid cancer. Our data support the suggested relationship with dietary fish or other marine products and may, at least partly, explain the long-lasting south–north risk gradient being present in the Norwegian population. Further investigation is needed to reveal the specific underlying factors.

### REFERENCES

Akslen L, Nilssen S and Kvåle G (1992) Reproductive factors and risk of thyroid cancer. A prospective study of 63,090 women from Norway. *Br J Cancer* **65**: 772–774

Ballivet S, Salmi LR, Dubourdieu D and Bach F (1995) Incidence of thyroid cancer in New Caledonia, South Pacific, during 1985–1992. *Am J Epidemiol* **141**: 741–746

Berg J, Glattre E, Haldorsen T, Høstmark A, Bay I, Johansen A and Jellum E (1994) Longchain serum fatty acids and risk of thyroid cancer: a population-based case–control study in Norway. *Cancer Causes Control* **5**: 433–439

© Cancer Research Campaign 1997

Central Bureau of Statistics of Norway (1971) *Norwegian Official Statistics A 399. Labour market statistics 1970*. Central Bureau of Statistics of Norway: Oslo, Norway

Central Bureau of Statistics of Norway (1976) *Occupational mortality 1970–1973*. Central Bureau of Statistics of Norway: Oslo, Norway.

Directorate of Labour (1965) *Standard classification of occupations in Norwegian Official Statistics* (in Norwegian). Directorate of Labour: Olso

Fox A and Adelstein A (1978) Occupational mortality: work or way of life? *J Epidemiol Commun Hlth* **32**: 73–78

Franceschi S, Levi F, Negri E, Fassina A and La Vecchia C (1991) Diet and thyroid cancer: a pooled analysis of four European case–control studies. *Int J Cancer* **48**: 395–398

Fugelli P, Tandberg A, Trygg K, Lund-Larsen K and Østgård L (1987) Diet and consumption of stimulants among 128 North Cape fishermen (in Norwegian). *Tidsskr Nor Laegeforen* **107**: 1741–1745

Glattre E, Finne TE, Olesen O and Langmark F (1985) *Atlas of cancer incidence in Norway 1970–1979*. The Cancer Registry of Norway/Norwegian Cancer Society: Oslo, Norway

Glattre E, Akslen LA, Thoresen S and Haldorsen T 1990*a* Geographic patterns and trends in the incidence of thyroid cancer in Norway 1970–1986. *Cancer Detect Prev* **14**: 625–631

Glattre E, Høstmark A, Thoresen SØ, Smith AJ and Akslen LA (1990*b*) Provocation of thyroid neoplasms in female Wistar rats fed N-3 or N-6 fatty acids in the feed. *Thyroidology* **2**: 1–3

Glattre E, Haldorsen T, Berg JP, Stensvold I and Solvoll K (1993) Norwegian case–control study testing the hypothesis that seafood increases the risk of thyroid cancer. *Cancer Causes Control* **4**: 11–16

Hands E (1990) *Food finder: food sources of vitamins and minerals*. ESHA Research: Salem, Oregon.

Kanno J, Onodera H, Furuta K, Maekawa A, Kasuga T and Hayashi Y (1992) Tumor-promoting effects of both iodine deficiency and iodine excess in the rat thyroid. *Toxicol Pathol* **20**: 226–235

Kolonel L, Hankin J, Wilkens L, Fukunaga F and Ward Hinds M (1990) An epidemiologic study of thyroid cancer in Hawaii. *Cancer Causes Control* 223–233

Langsteger W, Koltringer P, Wolf G, Dominik K, Buchinger W, Binter G, Lax S and Eber O (1993) The impact of geographical, clinical, dietary and radiation-induced features in epidemiology of thyroid cancer. *Eur J Cancer* **29A**: 1547–1553

Levi F, Franceschi S, Gulie C, Negri E and La-Vecchia C (1993) Female thyroid cancer: the role of reproductive and hormonal factors in Switzerland. *Oncology* **50**: 309–315

McTiernan A, Weiss N and Daling J (1984) Incidence of thyroid cancer in women in relation to reproductive and hormonal factors. *Am J Epidemiol* **120**: 423–435

National Nutrition Council (1985) *Food in Norway – figures and facts* (in Norwegian). National Nutrition Council: Oslo, Norway

Opdahl S (1988) *Nutrition tables 1977–1985*. Central Bureau of Statistics of Norway: Oslo, Norway

Parkin D, Muir C, Whelan S, Gao Y, Ferlay J and Powell J (1992) Cancer incidence in five continents. Vol. 6. In *IARC Scientific Publication*, no. 120, (eds Parkin DM, Muir CS, et al). International Agency for Research on Cancer: Lyon, France

Pedersen E (1956) Thyroid cancer in Norway (in Norwegian). *Nord Med* **56**: 1108–1110

Pedersen E and Hougen A (1969) Thyroid cancer in Norway. In *Thyroid Cancer*, Hedinger, C. (ed.), UICC Monograph series Vol. 12. Springer: Berlin

Preston D, Lubin J, Pierce D and McConney M (1993) *Epicure User's Guide*. HiroSoft International: Seattle, WA, USA

Preston-Martin S, Jin F, Duda M and Mack W (1993) A case–control study of thyroid cancer in women under age 55 in Shanghai (People's Republic of China). *Cancer Causes Control* **4**: 431–440

Ron E, Kleinerman R, Boice J, Livolsi V, Flannery J and Fraumeni J (1987) A population based case–control study of thyroid cancer. *J Natl Cancer Inst* **79**: 1–12

Ron E, Lubin J, Shore R, Mabuchi K, Modan B, Pottern L, Schneider A, Tucker M and Boice JJ (1995) Thyroid cancer after exposure to external radiation: a pooled analysis of seven studies. *Radiat Res* **141**: 259–277

Salabe G (1994) Aetiology of thyroid cancer: an epidemiological overview. *Thyroidology* **6**: 11–19

Thoresen S, Glattre E and Johansen A (1986) Thyroid cancer in Norway 1970–1979. Regional variation of histological types (in Norwegian). *Tidsskr Nor Laegeforen* **106**: 2616–2620

Williams E, Doniach I, Bjarnason O and Michie W (1977) Thyroid cancer in an iodide rich area. *Cancer* **39**: 215–222

© Cancer Research Campaign 1997